# Experimental Demonstration of Masking Phenomena between Competing Odorants via an Air Dilution Sensory Test

**DOI:** 10.3390/s100807287

**Published:** 2010-08-03

**Authors:** Ki-Hyun Kim

**Affiliations:** Atmospheric Environment Laboratory, Department of Environment & Energy, Sejong University, Seoul, 143-747 Korea; E-Mail: khkim@sejong.ac.kr; Tel.: +82-2-3408-3233; Fax: +82-2-3408-4320

**Keywords:** human sensing, odor masking, threshold, hydrogen sulfide, acetaldehyde, dilution-to-threshold (D/T) ratio

## Abstract

To simulate the occurrence of masking phenomena with the aid of an air dilution sensory (ADS) test, two types of odorant mixtures were prepared: (1) M_2_ with two individual odorants [H_2_S and acetaldehyde (AA)] and (2) M_6_ with six individual odorants (H_2_S and five aldehydes). The test results derived for samples containing single individual odorants at a wide range of concentrations are initially used to define the empirical relationship between the dilution-to-threshold (D/T) ratio and odor intensity (OI) scaling. Based on these relationships, the D/T ratios were estimated for each odorant with the same intensity as the synthetic mixture. The relative contribution of each odorant to such mixture is then assessed by comparing the estimated and measured D/T values. This stepwise test confirmed the dominance of certain compounds at a given OI rating. In the case of M_2_, H_2_S showed sensitive detection at high OI range, while AA did so at low end. The pattern of a competing relationship is also seen consistently from M_6_ between AA (low) and *iso*-valeraldehyde (IA: high OI range). The overall results thus suggest that the masking phenomena between strong odorants should proceed under competing relationships, if released at the same time.

## 1. Introduction

Malodorous compounds can be classified as an airborne pollutant group that can create a nuisance through irritation of the nasal sensory system. Although odorants are produced by diverse source processes, many of them tend to share strong similarities such as high volatilities with significantly low threshold values. Consequently, their presence above the threshold levels can be considered the cause of mental or psychological stress and as likely potential threats to humans and ecological systems.

In light of the diverse properties each odorant has, the process of synthetic mixing is one of the most puzzling issues in odor research in terms of the intensity scaling [1]. If the synthetic mixing of multiple odorants takes place, sensory inputs are expected to arise for each individual component and/or their mixtures [2]. The resulting odor intensity can then be postulated either as enhancement via synergism or diminishment via suppression. In reality, however, the perception of individual components or assessment of their relative roles can be restricted to a large extent by the mixing process because of the non-linear interactions in sensory cell responses [2,3]. Enormous efforts devoted to behavioral studies in fact helped us confirm indirectly the effect of synthetic mixing such as hyperadditivity or odor masking [4]. Early studies of synthetic mixing commonly relied on tests with animals or insects such as rats [5], honeybees [2], and lobsters [6]. These physiological studies on sensory representations suggest that odor masking can proceed, since the odor intensity of the two (or multiple) odorant mixtures is weaker than that expected from their sum [7]. Nonetheless, to a large extent, the results of such qualitative assessment on mixing effect cannot yet overcome the barriers to a systematic and quantitative description of complex odor [1].

At present, quantitative analysis of malodor depends on both direct (olfactometry) and indirect (instrumental) methods [8]. As one good example of the former approaches, an air dilution sensory (ADS) test can be performed through a quantification of the dilution-to-threshold (D/T) ratio of samples at whose level of dilution the odor threshold is recognized [8]. This type of direct method can be exercised without the employment of expensive equipment for measurements. Fortunately, the reliability of these approaches has gradually been improved through continuous modifications to yield fairly objective results [8]. In contrast, the use of an indirect method can be optimized to provide the common concentration data of individual odorants with great reproducibility and accuracy with the aid of advanced technology [8]. Although both methods can target the identical samples, the results of both analyses can be linked only in an indirect manner. For instance, the odor intensity (OI) of a given sample can be accessed through the conversion from its concentration value with the application of empirical equations [9,10].

In this research, a series of laboratory tests have been conducted to measure the D/T ratios of standard samples containing both individual odorants and their mixtures, as a basic tool to describe the fundamental aspects of synthetic mixing between multiple odorants. The experimental data derived from individual odorants were then used to provide a quantitative assessment of synthetic mixing processes between different odorants through a comparison of the measured and estimated D/T ratios. Based on this comparative analysis, the occurrence pattern of masking phenomena is explained in terms of relative dominance of the individual odorant at a given OI range.

## 2. Materials and Methods

### 2.1. Experimental Scheme for Masking Effects

In this study, the effects of synthetic mixing were investigated using six individual odorants consisting of one reduced sulfur compound (H_2_S) and five aldehydes [acetaldehyde (AA), propionaldehyde (PA), butyraldehyde (BA), *iso*-valeraldehyde (IA), and valeraldehyde (VA)]. The general characteristics of these target odorants are described in Table 1. These compounds were selected because of their significance as the main offensive odorants according to the Korean malodor prevention law [11]. Most of them are in fact well-known to be listed as low threshold-level odorants released from strong anthropogenic sources such as industrial processing or sewage treatment processing [12–14].

**Figure 1.**
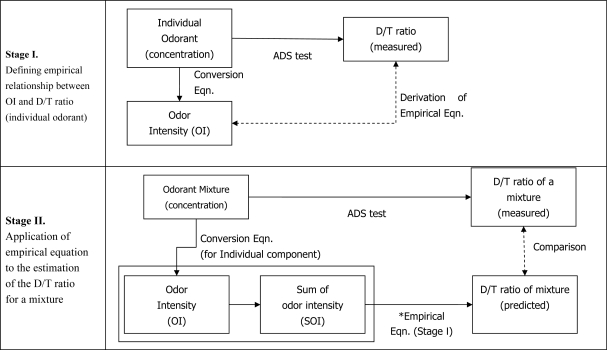
Schematic of the two-stage approaches for (1) the estimation of empirical relationships between D/T ratio and odor intensity of individual odorants and (2) the application of such relationships to mixed odorants.

The basic experimental scheme of our study is presented in Figure 1. The major components of this study can be divided into the two stages. In the first stage, the relationships between different expression units for odor composition are defined between pre-existing knowledge or methods.

**Figure 2.**
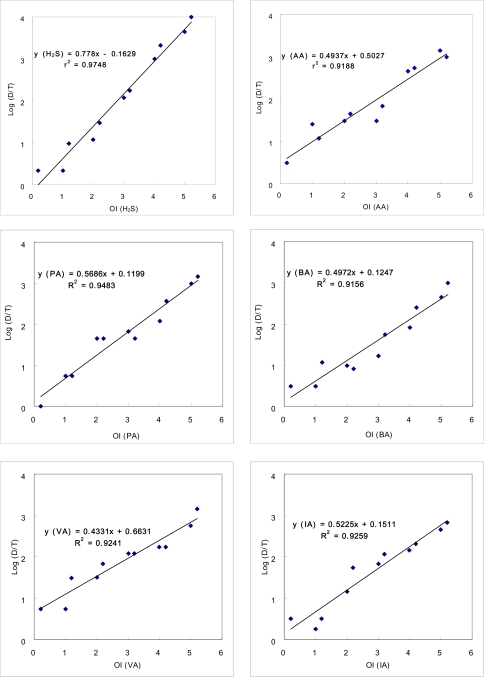
Relationship between odor intensity and dilution-to-threshold (D/T) ratio derived for six target compounds.

For instance, odor intensity is first calculated from the known concentrations of each odorant whose samples are prepared at 11 OI ratings (refer to Nagata [10]). These individual samples are then subject to the ADS test to assign the corresponding D/T ratios. Then, by combining these OI and D/T ratios, one can now newly establish empirical equations to describe their relationships (Figure 2). These newly established equations from the stage 1 experiment is then used to estimate the D/T ratios of a given compound contained in the M_2_ or M_6_ samples at the next stage. Hence, information concerning these estimated D/T results for each of all individual components in a mixture provides the very basic tools to interpret and estimate its relative contribution to various mixture samples.

### 2.2. The Preparation of Malodor Samples

For the purpose of our study, odorant samples for the ADS test were prepared based on two different criteria. In the first stage of the ADS experiment, each odorant sample was prepared using the both respective standard gases of H_2_S and aldehydes (Ri Gas, Korea). In order to define empirical relationships of individual odorants between their concentration levels (or OI) and D/T ratios, samples were prepared to match a wide range of odor intensities (*i.e.*, 11 levels in this study) that end in one decimal point with either 0 or 2 such as 0.2, 1.0, 1.2, 2.0, and so on (Table 2).

To comply with our experimental scheme in the second stage, two distinct approaches were used for the preparation of odor mixture samples such as two (M_2_) and six odorants (M_6_). The M_2_ samples were made by mixing H_2_S and AA, while the M_6_ samples were made by mixing H_2_S and all five aldehydes (Table 3). The concentration levels of individual components used for each sample of the M_2_ mixture were basically assigned to maintain a comparable range of odor intensities. For instance, according to the equation defining the relationship between odor concentration and OI [10], the two components of the first M_2_ sample has the concentrations of 0.35 ppb (H_2_S) and 1.0 ppb (AA) to maintain their OI values near 0.8 (Table 3). In compliance with this criterion, the ninth M_2_ sample was made up of 3,500 ppb H_2_S (OI = 4 .66) and 9,960 ppb AA (OI = 4.86).

In the case of the M_6_ samples, a slight modification was applied in their preparation to facilitate the mixing of more complicated compositions at a total of nine different OI levels. To this end, a pre-mixture of five aldehyde standards was mixed with the H_2_S standard gas. To allow a parallel comparison between M_2_ and M_6_ samples, the concentrations of H_2_S and AA in all M_6_ samples were assigned identically to those of the M_2_ (Table 4).

The concentrations of all the other aldehydes were assigned accordingly, as the pre-mixture had a fixed composition with the inter-odorant molar ratios of 1.0 (AA): 0.2 (PA): 0.19 (BA): 0.15 (VA): 0.20 (IA). This enhanced level of AA in the standard five aldehyde gas mixture was necessary to compensate for its significantly reduced OI levels relative to others. However, as the OI levels of different aldehydes at the equal concentration level can be greatly differentiated by the conversion formula of Nagata, the OI values of each aldehyde component in the first M_6_ sample exhibited several-fold differences: 0.13 of PA (0.20 ppb) to 1.0 of IA (0.20 ppb). Likewise, the OI values of the ninth M_6_ sample also changed from as little as 4.17 (2,010 ppb PA) to 6.40 (1,960 ppb IA). Despite this superior position of IA in all the M_6_ mixture compositions, AA still took the role of the most dominant contributor in the estimated D/T levels at the early stage of the M_6_ samples (Table 4). Overall, this pronounced pattern of mixture composition (*i.e.,* predominance of IA in terms of OI) indeed helped us differentiate the actual pattern of masking phenomenon from many competing odorants in relation to both H_2_S and other aldehydes.

### 2.3. Air Dilution Sensory (ADS) Test Based on Olfactometry Threshold Method

In this work, actual application of the air dilution sensory (ADS) test was carried out according to the standard procedure established by the Korean Ministry of the Environment (KMOE). The KMOE method of the ADS test belongs to a threshold olfactometry in which the central trend of the odor index value is derived geometrically for a given odor sample, after excluding the data sets of two extreme ends obtained from each round of the test. Developed and modified from the triangle odor bag method of Japan [9], it is currently the main test method in Korea. The samples prepared either individually or as mixtures were then subject to the ADS test by a panel of five members; all of these members were selected based on a pre-screening test in which all participants are requested to differentiate samples of deionized water from testing solutions containing four chemicals with the following weight (%): acetic acid (1), TMA (0.1), methylcyclopentenolone (3.2), and phenethyl alcohol (1).

The static dilution of odorant samples for the ADS test was made in a stepwise manner by mixing original odorant samples with odorless air using a 3 L odor bag made of polyethylene telephtalate film. Odorless air was prepared by passing normal air into an activated charcoal filter. The ADS test was conducted continuously using odorant samples prepared through a stepwise dilution. This test was completed, when the last panel member reached the minimum detection (threshold values) of a given odor sample. The level of dilution for the ADS test progressed through an application of the multiplying factors derived as X values: X= Z 10^n^, where, the superscripted value ‘n’ corresponds to an integer value of 0, 1, 2, 3,…., n. In addition, Z is a multiplying factor of either 1 or 3. The odor index value for a given sample is then processed by the stipulated method of KMOE [11]. The results of the 2-stage ADS experiments are ultimately expressed as D/T ratios through a combination of the ‘yes/no’ opinions from all panel members.

## 3. Results and Discussion

### 3.1. Relationships between the Concentrations of Individual Odorant *vs*. the Corresponding D/T Ratios

As the first step of our study, the relationship between the concentrations of individual odorant *vs.* the corresponding D/T ratios was examined by assigning the D/T ratios measured from the ADS test to the known concentrations (or converted OI values) of odorant samples. The results of the ADS test for each individual odorant, expressed in terms of D/T ratios, are presented in Table 2 for a total of 11 samples. In the case of the H_2_S, the sample with the lowest OI value of 0.2 (or 0.07 ppb in concentration) yielded a D/T ratio of 2, while the sample with the maximum OI of 5.2 recorded a D/T ratio of 10,000. Hence, the concentration ratio of H_2_S between the maximum and minimum (13,060/0.07 = 1.9 × 10^5^) is clearly distinguishable from its D/T counterpart (10,000/2 = 5 × 10^3^). If this comparison is extended to aldehydes, these ratio values are computed at much reduced levels. For instance, their corresponding ratio values for AA are reduced by more than an order of magnitude to 9 × 10^4^ (=21,700/0.24) and 3.3 × 10^2^ (=1,000/3), respectively. According to this comparison, H_2_S exhibits a relatively large slope value of 0.78 with a small negative offset value. In contrast, all aldehydes consistently share strong similarities with slope values near 0.5 and positive offset values ranging between 0.12 (PA) and 0.66 (VA). Hence, changes in the D/T ratio tend to proceed much rapidly for H_2_S across the entire OI range relative to AA or other aldehydes. In other words, human perception of H_2_S can occur more dynamically than that of aldehydes. Likewise, differences in human perception pattern can be meaningfully distinguished between different odorants, if the relationships are assessed between OI and log D/T ratios (Figure 1). In addition, the relationship between OI and D/T ratios is further distinguished by the magnitude of coefficient of determination, r^2^. Although r^2^ value of H_2_S is high enough to show 0.97, those for aldehydes tend to vary in slightly reduced values of 0.92 to 0.95. This finding thus indicates that the olfactory detection of H_2_S can be made in a more predictable and systematic manner than those for aldehydes. The basic characteristics of individual odorants can therefore be accounted for at least partially by the interactive relationship between their slope and offset values.

### 3.2. Estimation of D/T Ratios for Odorant Mixture

As explained above, the relationship between odorant concentrations and all the related parameters (e.g., odor intensity and D/T ratio) can be defined basically for any of the individual odorants. In contrast, the evaluation of intensity for an odorant mixture becomes a more complicated task than that for a particular odorant odor. In order to simplify the assessment of the OI levels for odorant mixtures like M_2_ or M_6_ samples, the sum of odor intensity (SOI) concept was applied to each mixture sample by binding the OI values of individual odorants in a logarithmic scale e.g., [8] as follows:

$${\text{SOI}}_{i}=\text{log}(10^{\text{OI}(i)1}+10^{\text{OI}(i)2}+10^{\text{OI}(i)3}+\cdots +10^{\text{OI}(i)n})$$

where OI(i) = odor intensity of individual odorant in the “i”th stage standard mixture. The subscripts 1 through n correspond to the order of the individual components of the mixture. According to this conversion formula, the first M_2_ sample consisting of 0.35 ppb H_2_S and 1 ppb AA is computed to have an SOI value of 1.14 (Table 3). Likewise, the first M_6_ sample has the corresponding SOI value of 1.509, as shown in Table 4. Because SOI values can be assigned to any kind of mixture with a complicated composition, comparison of odor strengths for both types of odor mixtures (M_2_ and M_6_) in this study can be made on the parallel basis. In order to estimate the relative contribution of individual components to the strength of mixed odorants, the D/T ratios for mixture samples have been evaluated in a stepwise manner. It should first be noted that the D/T ratios for a single odorant can be calculated for the samples with any OI values through empirical equations, as defined by the empirical relationships (Figure 1). This type of approach used for individual odorant can now be extended further to predict the D/T ratios for the mixture. To initiate this estimation, a sample of odorant mixture with a given SOI value is assumed to be represented by any single constituent that has the equivalent OI level. If one considers the first M_2_ sample, it has a computed SOI value of 1.14 with a measured D/T ratio of 10^0^. The D/T ratios for this mixture sample can thus be first approximated by the single odorant with the same odor intensities. Under such an assumption, the odor intensity scaling of this mixture can be approximated by any single odorant (H_2_S or AA) with the identical strengths (*i.e.*, OI values of 1.14), which can yield the corresponding D/T ratios of either 10^0.5^ (H_2_S) or 10^0.91^ (AA) (Table 3B). This approach can also be applied to M_6_ samples in an identical manner. Hence, in the case of the first M_6_ sample with the SOI value of 1.51, its D/T ratios are estimated from six individual components with the identical OI value of 1.51. According to this approximation, the estimated D/T ratios for this M_6_ sample tend to fall in a much wider range of 10^0.19^ (PA) to 10^0.91^ (AA). Because each single component consisting of the synthetic mixture samples can make different contributions, the D/T values estimated for each individual component can later be used to assess their relative roles through a direct comparison with the measured D/T ratios.

### 3.3. Comparison of Estimated *vs*. Measured D/T Ratios

**Figure 3.**
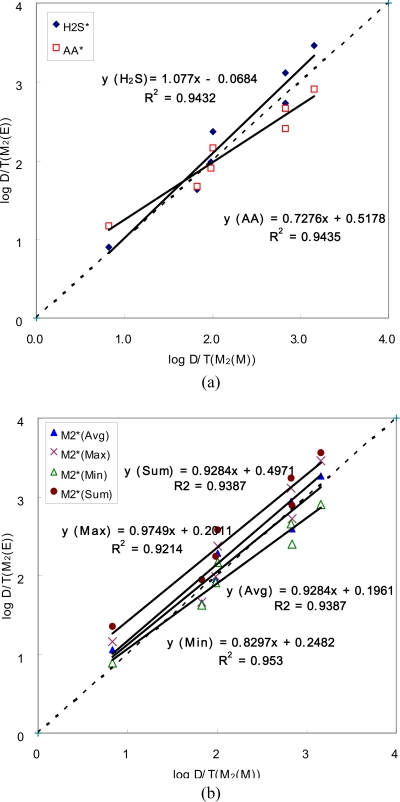
Comparison of the D/T ratios for M_2_ between measured (D/T(M_2_(M))) and estimated values with various combinations (D/T(M_2_(E))); (a) individual compound and (b) artificial combinations. Letters of M and E in the parenthesis denote measured and estimated, respectively.

**Figure 4.**
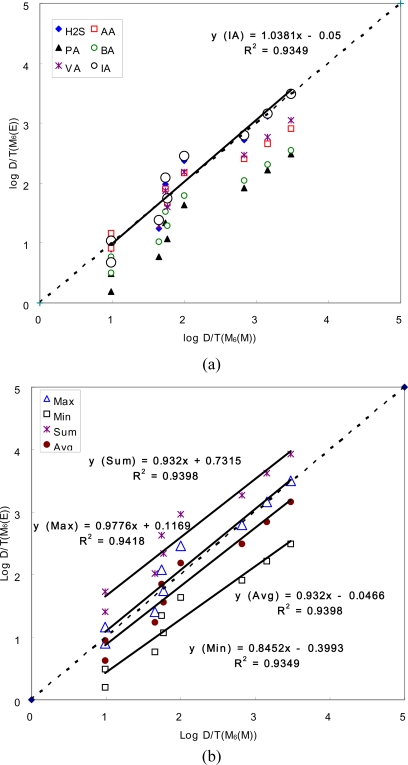
Comparison of the D/T ratios for M_6_ between measured (D/T(M_6_(M))) and estimated values with various combinations (D/T(M_6_(E))); (a) individual compound and (b) artificial combinations. Letters of M and E in the parenthesis denote measured and estimated, respectively.

As shown in Table 3, the D/T ratios of mixed odorants (M_2_) can be estimated roughly by the alternate single component through the empirical relationship and can be compared with the measured counterparts in a diverse manner. Because D/T ratios of the mixture, M_2_, are initially estimated from the two individual components, the actual measurement for M_2_ can be compared either with the direct estimates for individual components (H_2_S or AA) or with their indirect statistical derivatives (like maximum, minimum, sum, and average of the two). In Figures 3 and 4, comparisons between the estimated and measured D/T ratios are made for M_2_ and M_6_ samples, respectively.

The pattern derived for the M_2_ sample indicates that AA is dominant over H_2_S in lower OI range, although it is reversed in upper range. However, if the effect of M_2_ mixing has to be represented by any single component within the mixture, H_2_S appears to mimic the behavior of M_2_ in a more comparable manner in terms of the D/T ratio (note that H_2_S shows a slope value near unity with a small negative offset value). To learn more about the interactive roles between different components in the mixture, the evaluation of the M_2_ data can be extended further to those of diverse derivatives extracted from the two individual components. As shown in Figure 3b, the best compatibility between the two types of D/T values is recognized by the maximum components between the two individual odorants which exhibit the slope values approaching the unity (0.97) with the least offset value of 0.20.

The results extracted from derivative components thus suggest the possibility that the odor strength of the mixture should be represented by the single component dominant at a given OI range, if the property of such mixture has to be determined by the combined effects of all individual odorant components rather than by single component. In a qualitative sense, identification of single components in odorant mixture becomes more complicated with an increasing number of odorants due to the limited configurational capacity of human olfaction [15]. However, as shown below, from the standpoint of the overshadowing effect via threshold detection, the prominence of certain odorant(s) can be identified more definitively in the mixtures with the complicated compositions. As the D/T ratios for the M_2_ samples were compared in Table 3, those of M_6_ samples are also assessed in Table 4. As explained above, the measured D/T ratios for the M_6_ samples can be compared directly with each of all six individual components as well as with those derivatized statistically from the individual ones. The comparison of the data derived from this complicated mixture indicates that the competing relationship holds between different odorants across varying OI scalings. As shown in Table 4, comparison of the estimated log(D/T) values indicates that IA is the predominant component over most of the upper OI range, while the mixture can be represented by AA in a short range of lower OI values. As a result, the measured D/T data, when compared with the estimates of the individual components, are best accounted for by those of IA (Figure 4a).

However, if the measured D/T ratios have to be explained by the combined effects of all competing components such as the ones derivable from the statistical modification, the best compatibility is again attained by the maximum D/T ratios among all six competing components across samples of increasing intensity scale. In the case of the M_2_ sample, evaluation of the compatibility between different statistical parameters was slightly complicated between the measured and estimated D/T values, as the former showed good similarities to those derived as either average or maximum (Figure 3b). However, in the case of the M_6_ sample, the representativeness of the maximum component becomes more evident than any other derivatives (sum or average), despite the fact that M_6_ has more complicated composition than that of M_2_ (Figure 4b). The results of this comparative analysis thus show a good agreement with the general definition of hyperadditivity or masking in that mixtures of two (or more) odorants are less intense than would be predicted from their sum [5]. The direction of the present study also appears to comply with previous efforts to search the rules of dose additivity using such binary mixture pairs as 1-butanol and 2-heptanone [16] or toluene and butyl acetate [17]. These authors found that the effect of dose-addition diminishes noticeably with increasing level of addition. Thus, they conclude that olfactory sensation has a more selective window of chemical tuning for odorant mixture than does trigeminal chemoreception [1].

## 4. Conclusions

In this work, a series of laboratory experiments were conducted to assess the basic aspects of synthetic mixing between different odorants. To this end, we first examined the relationships between the odor intensities of independent odorants through the air dilution sensory (ADS) test in terms of the dilution-to-threshold (D/T) ratio. The results of these initial experiments were further used to estimate the D/T ratios for diverse odorant mixtures consisting of both two (M_2_) and six individual components (M_6_) over a wide range of odor intensities. The effect of synthetic mixing was then evaluated through a comparative analysis between estimated and measured D/T ratios for both types of mixture samples. The results of our comparative analysis between different odorant samples consistently indicate that the odor intensity of a mixture is determined by the component that is dominant at a given OI range. If the estimated D/T ratios of individual components are put together to yield diverse statistical derivatives, the maximum D/T component derived among all individual constituents at a given OI range coincides most effectively with the measured D/T ratios. As a result, the threshold level of the odorant mixtures is subject to the intensity of such single predominant component that is most representative at a given OI range rather than the sum or average of all individual ones. Overall, the direct quantification of D/T ratios by the human test panel was helpful in demonstrating the occurrence of the masking phenomenon between different odorants from complex mixture combinations established by a number of well-defined offensive odorants. Future study is thus recommended to extend our initial efforts through simulation of more complicated conditions with diverse odorant compositions.

## Figures and Tables

**Table 1.** List of target compounds investigated for relationship with dilution-to-threshold (D/T) ratio.

**Order**	**Carbonyl compound**	**Short name**	**Molecular formula**	**CAS number**	**Chemical structure**	**Molecular weight**
1	Hydrogen sulfide	-	H_2_S	7783-06-04	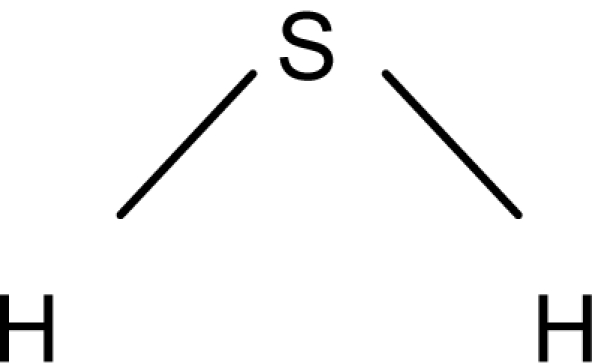	34
2	Acetaldehyde	AA	CH_3_CHO	75-07-0	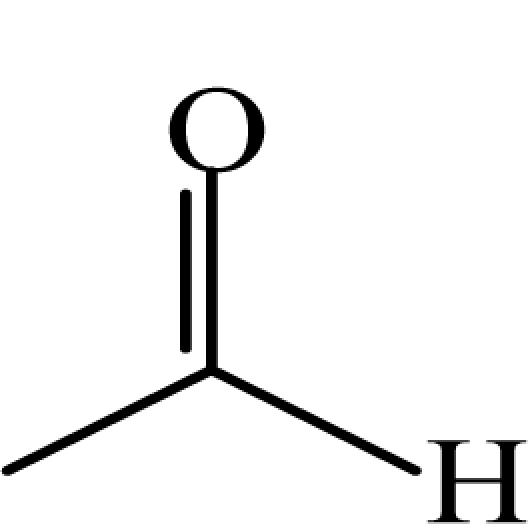	44.1
3	Propionaldehyde	PA	CH_3_CH_2_CHO	123-38-6	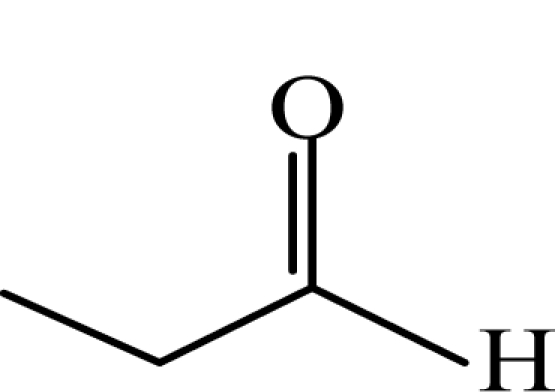	58.1
4	Butyraldehyde	BA	CH_3_CH_2_CH_2_CHO	123-72-8	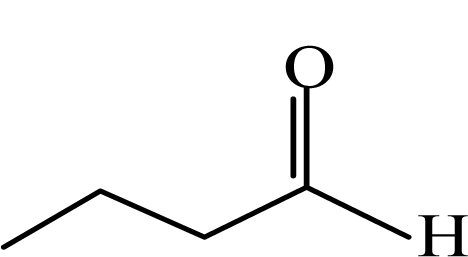	72.1
5	Iso-valeraldehyde	IA	(CH_3_)_2_CHCH_2_CHO	590-86-3	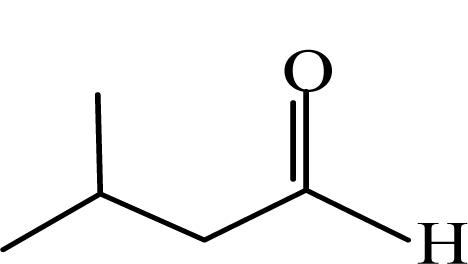	86.1
6	Valeraldehyde	VA	CH_3_(CH_2_)_3_CHO	110-62-3	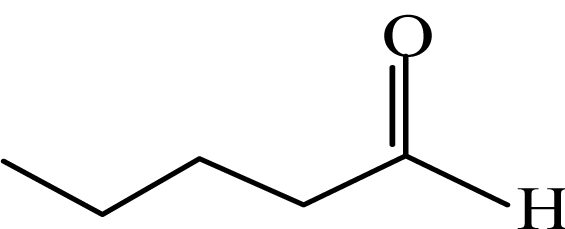	86.1

**Table 2.** Preparation of gaseous standards for each individual odorant to cover a wide range of odor intensities and results of dilution-to-threshold measurements.

**Order**	**Odor intensity (OI)^a^**	**Target odorant**					
		**H_2_S**	**AA**	**PA**	**BA**	**VA**	**IA**
		[A] Concentration (ppb)					

1	0.2	0.07	0.24	0.24	0.05	0.18	0.05
2	1.0	0.50	1.50	1.47	0.31	0.71	0.19
3	1.2	0.80	2.39	2.32	0.49	1.00	0.27
4	2.0	5.59	14.7	14.4	2.92	3.87	1.07
5	2.2	9.08	23.2	22.6	4.57	5.44	1.51
6	3.0	63.1	144	142	27.3	21.1	5.89
7	3.2	102	227	222	42.8	29.6	8.29
8	4.0	710	1,410	1,376	256	115	32.5
9	4.2	1,160	2,220	2,171	400	161	45.6
10	5.0	8,040	13,740	13,457	2,391	623	178
11	5.2	13,060	21,700	21,218	3,751	872	251

		[B] The resulting dilution-to-threshold (D/T) ratio ^b^					

1	0.2	2.15	3.11	1.00	3.11	5.48	3.11
2	1.0	2.15	25.4	5.48	3.11	5.48	1.76
3	1.2	9.65	11.8	5.48	11.8	30.5	3.11
4	2.0	11.8	30.0	44.8	9.65	31.1	14.4
5	2.2	30.0	44.8	44.8	8.18	66.9	54.8
6	3.0	118	30.0	66.9	17.0	118	66.9
7	3.2	173	66.9	44.8	54.8	118	116
8	4.0	1,000	448	120	81.8	173	142
9	4.2	2,080	548	367	250	173	208
10	5.0	4,481	1,390	1,000	448	557	448
11	5.2	10,000	1,000	1,442	1,000	1,442	669

a Functional formulas are used to convert concentrations of individual odorants into odor intensity (OI) based on the empirical functions in the reference [10]: Y (H_2_S) = 0.950 logX + 4.14; Y (AA) = 1.010 logX + 3.85; Y (PA) = 1.010 logX + 3.86; Y (BA) =1.030 logX + 4.61; Y (VA) = 1.360 logX + 5.28; and Y (IA) = 1.350 logX + 6.01. Here, odor intensity (Y) is derived by inserting concentration values (X) in ppm unit.

b Denotes the dilution-to-threshold (D/T) ratio determined by a five member odor testing panel.

**Table 3.** Relationship between odor intensity and dilution-to-threshold (D/T) ratios for an odorant mixture consisting of two individual compounds (M_2_).

A. Derivation of odor intensity for gaseous mixtures of two individual odorants.					

**Order**	**Concentration (ppb)**		**OI/SOI^a^**		

	**H_2_S**	**AA**	**H_2_S**	**AA**	**M_2_^b^**
1	0.35	1.00	0.85	0.82	1.14
2	1.18	3.32	1.36	1.35	1.65
3	3.48	10.0	1.80	1.83	2.12
4	11.7	33.2	2.30	2.36	2.63
5	35.0	99.6	2.76	2.84	3.10
6	117	332	3.25	3.37	3.61
7	350	996	3.71	3.85	4.08
8	1,170	3,320	4.20	4.38	4.60
9	3,500	9,960	4.66	4.86	5.07

a For the determination of odor intensity (OI) for individual compounds, Nagata's empirical formula [10] was employed. In the case of a two-compound mixture (M_2_), the sum of odor intensity (SOI) was derived as: SOI = log(10^OI(a)^ + 10^OI(b)^);

b M_2_ denotes the mixture of two compounds at each of all 9 concentration levels.

**Table:** 

B. Comparison of odor intensity with various D/T ratios.								

**Order**	**OI/SOI^a^**	**Log (D/T)**						
		**M_2_(M)^b^**	**H_2_S(E)^c^**	**AA(E)**	M_2_*(E-Max)^d^	M_2_*(E-Min)	M_2_*(E-Sum)	M_2_*(E-Avg)
1	1.14	0.00	0.50	0.91	0.91	0.50	1.05	0.75
2	1.65	0.83	0.89	1.17	1.17	0.89	1.35	1.05
3	2.12	0.49	1.24	1.41	1.41	1.24	1.63	1.33
4	2.63	1.83	1.63	1.67	1.67	1.63	1.95	1.65
5	3.10	1.98	1.98	1.90	1.98	1.90	2.25	1.94
6	3.61	2.00	2.37	2.16	2.37	2.16	2.58	2.28
7	4.08	2.83	2.72	2.40	2.72	2.40	2.89	2.59
8	4.60	2.83	3.11	2.66	3.11	2.66	3.24	2.94
9	5.07	3.16	3.46	2.90	3.46	2.90	3.57	3.26

a Same as explained above.

b, c M and E denote ‘measured’ and ‘estimated’, respectively.

d Asterisks (*) denote that D/T ratios are estimated by taking max, min, sum, and average values from two components of a mixture (H_2_S and AA data).

**Table 4.** Preparation of odor mixture consisting of six compounds (M_6_) and the relationship between odor intensity and dilution-to-threshold (D/T) ratio^a^.

A. Detailed information of individual odorants added for a mixture odorant of M_6_.						

**Order**	**Individual compound**					
	**H_2_S**	**AA**	**PA**	**BA**	**VA**	**IA**
[1] Concentrations of odorants used for the mixed standards (ppb)						

1	0.35	1.00	0.20	0.19	0.15	0.20
2	1.18	3.32	0.67	0.62	0.50	0.65
3	3.48	10.0	2.01	1.86	1.51	1.96
4	11.7	33.2	6.70	6.20	5.03	6.53
5	35.0	99.6	20.1	18.6	15.1	19.6
6	117	332	67.0	62.0	50.3	65.3
7	350	996	201	186	151	196
8	1,170	3,320	670	620	503	653
9	3,500	9,960	2,010	1,860	1,510	1,960

[2] Odor intensity (OI) of the above-listed individual odorants added for M_6_						

1	0.85	0.82	0.13	0.77	0.08	1.00
2	1.36	1.35	0.65	1.31	0.79	1.71
3	1.80	1.83	1.14	1.80	1.44	2.35
4	2.30	2.36	1.66	2.34	2.15	3.06
5	2.76	2.84	2.15	2.83	2.80	3.70
6	3.25	3.37	2.67	3.37	3.51	4.41
7	3.71	3.85	3.16	3.86	4.16	5.05
8	4.20	4.38	3.68	4.40	4.87	5.76
9	4.66	4.86	4.17	4.89	5.52	6.40

[3] log (D/T) estimates for individual odorants based on their relationship with OI						

1	0.50	**0.91**	0.19	0.51	0.70	0.68
2	0.89	**1.17**	0.49	0.77	1.01	1.04
3	1.24	**1.41**	0.77	1.02	1.29	1.38
4	1.63	1.67	1.07	1.29	1.60	**1.75**
5	1.98	1.90	1.34	1.53	1.88	**2.09**
6	2.37	2.16	1.64	1.80	2.19	**2.46**
7	2.72	2.40	1.91	2.04	2.47	**2.79**
8	3.11	2.66	2.21	2.31	2.77	**3.16**
9	3.46	2.90	2.49	2.55	3.06	**3.50**

B. Relationship between the sum of odor intensity and log (D/T) ratios: the latter derived for comparison between the actual measurements (M_6_(M)) and the prediction terms (M_6_*(E-)).						

	**SOI**	**M_6_(M)**	M_6_*(E-Max)^c^	M_6_*(E-Min)	M_6_*(E-Sum)	M_6_*(E-Avg)
1	1.509	0.98	0.91	0.19	1.41	0.63
2	2.105	0.98	1.17	0.49	1.72	0.94
3	2.664	1.65	1.41	0.77	2.01	1.23
4	3.297	1.77	1.75	1.07	2.33	1.56
5	3.891	1.74	2.09	1.34	2.63	1.85
6	4.555	2.00	2.46	1.64	2.97	2.19
7	5.172	2.83	2.79	1.91	3.27	2.50
8	5.858	3.16	3.16	2.21	3.62	2.84
9	6.489	3.48	3.50	2.49	3.94	3.16

a Refer to Table 2 for all comparable notations.
